# Microstructure, Mechanical and Tribological Properties of Advanced Layered WN/MeN (Me = Zr, Cr, Mo, Nb) Nanocomposite Coatings

**DOI:** 10.3390/nano12030395

**Published:** 2022-01-26

**Authors:** Kateryna Smyrnova, Martin Sahul, Marián Haršáni, Alexander Pogrebnjak, Volodymyr Ivashchenko, Vyacheslav Beresnev, Vyacheslav Stolbovoy, Ľubomír Čaplovič, Mária Čaplovičová, Ľubomír Vančo, Martin Kusý, Alexey Kassymbaev, Leonid Satrapinskyy, Dominik Flock

**Affiliations:** 1 Department of Nanoelectronics and Surface Modification, Sumy State University, Rymskogo-Korsakova 2, 40007 Sumy, Ukraine; alexp@i.ua; 2 Institute of Materials Science, Slovak University of Technology in Bratislava, Jána Bottu 25, 917 24 Trnava, Slovakia; martin.sahul@stuba.sk (M.S.); lubomir.caplovic@stuba.sk (Ľ.Č.); martin.kusy@stuba.sk (M.K.); 3 Research and Development Department, Staton, s.r.o., Sadová 1148, 038 53 Turany, Slovakia; harsani@staton.sk; 4 Department of Biotechnology, Al-Farabi Kazakh National University, Almaty 050040, Kazakhstan; 5 Center of Advanced Development “VERITAS”, D. Serikbayev East Kazakhstan State Technical University, Protozanova 69, Ust-Kamenogorsk 070004, Kazakhstan; alexey_kasymbayev@mail.ru; 6 Institute for Problems of Material Science, NAS of Ukraine, Krzhizhanovsky 3, 03142 Kyiv, Ukraine; Ivashchenko@icnanotox.org; 7 Department of Materials for Reactor Building and Physical Technologies, V.N. Karazin Kharkiv National University, Svobody Sq. 4, 61022 Kharkiv, Ukraine; v.beresnev@karazin.ua; 8 Laboratory of Development and Research of Intensive Ion-Plasma Technologies, National Science Center Kharkiv Institute of Physics and Technology, Akademichna 1, 61108 Kharkiv, Ukraine; stolbovoy@kipt.kharkov.ua; 9 Centre for Nanodiagnostics of Materials, Slovak University of Technology in Bratislava, Vazovova 5, 812 43 Bratislava, Slovakia; maria.caplovicova@stuba.sk (M.Č.); lubomir.vanco@stuba.sk (Ľ.V.); 10 Department of Experimental Physics, Comenius University in Bratislava, Mlynská dolina F2, 842 48 Bratislava, Slovakia; leonid.satrapinskyy@fmph.uniba.sk; 11 Institute of Materials Science and Engineering, Ilmenau University of Technology, Gustav-Kirchhoff Str. 1, 98693 Ilmenau, Germany; Dominik.Flock@tu-ilmenau.de

**Keywords:** coating, microstructure, mechanical properties, wear, CA-PVD

## Abstract

Due to the increased demands for drilling and cutting tools working at extreme machining conditions, protective coatings are extensively utilized to prolong the tool life and eliminate the need for lubricants. The present work reports on the effect of a second MeN (Me = Zr, Cr, Mo, Nb) layer in WN-based nanocomposite multilayers on microstructure, phase composition, and mechanical and tribological properties. The WN/MoN multilayers have not been studied yet, and cathodic-arc physical vapor deposition (CA-PVD) has been used to fabricate studied coating systems for the first time. Moreover, first-principles calculations were performed to gain more insight into the properties of deposited multilayers. Two types of coating microstructure with different kinds of lattices were observed: (i) face-centered cubic (fcc) on fcc-W_2_N (WN/CrN and WN/ZrN) and (ii) a combination of hexagonal and fcc on fcc-W_2_N (WN/MoN and WN/NbN). Among the four studied systems, the WN/NbN had superior properties: the lowest specific wear rate (1.7 × 10^−6^ mm^3^/Nm) and high hardness (36 GPa) and plasticity index H/E (0.93). Low surface roughness, high elastic strain to failure, Nb_2_O_5_ and WO_3_ tribofilms forming during sliding, ductile behavior of NbN, and nanocomposite structure contributed to high tribological performance. The results indicated the suitability of WN/NbN as a protective coating operating in challenging conditions.

## 1. Introduction

Transition metal nitrides have become an essential part of manufacturing technologies. Due to the increased demands for drilling and cutting tools operating at extreme machining temperatures and rates, protective coatings are extensively utilized to prolong the useful tool life and eliminate the need for lubricants. Hence, they decrease the environmental hazards and increase the quality of fabricated tools, ultimately reducing maintenance costs. For many decades monolayer nitrides, such as TiN, ZrN, and CrN, have been used for tribological applications. However, numerous studies have demonstrated that alternating deposition of binary nitride layers produces multilayer coatings with superior properties, i.e., TiN/ZrN, TiN/CrN, CrN/ZrN, MoN/CrN, and TiN/MoN [1,2,3,4,5,6,7,8]. That resulted in the widespread interest in multilayer architecture since it provides high hardness, wear resistance, and thermal stability. On the one hand, it can effectively hinder crack propagation due to the numerous interfaces between layers and grain boundaries [9,10]. On the other hand, the multilayer concept unites properties specific to each individual layer in a new structure with unique and enhanced characteristics [11,12].

Among the various compositions of the binary multilayer nitride coatings, WN-based ones have not been comprehensively studied yet. However, WN exhibits high hardness, chemical stability, and tribological performance [13,14]. Despite this fact, only a few tungsten nitride multilayer systems combined with different transition metal nitrides were investigated. Duh et al. [15,16,17] and Li et al. [18] studied CrN/WN thin films prepared by magnetron sputtering and ion-beam assisted deposition. This system demonstrated similar results in all experiments. The tribological behavior was improved by introducing the ceramic/ceramic structure. For instance, the hardness of the multilayer system ranging between 28.6 and 30.5 GPa was superior to that of the individual layers: 24.3 GPa (WN) and 17.4 GPa (CrN) [16]. The incorporation of ZrN to the nanoscale WN-based multilayer coatings [19] resulted in an even higher hardness of about 34 GPa. Besides, the mechanical properties and oxidation behavior of TiN and WN were found to be enhanced by their combination in the multilayer architecture [20,21,22,23]. Hence, the current state of research demonstrates that WN-based multilayers are promising candidates for tribological applications. They should be able to withstand harsh dry machining and high-speed cutting conditions.

Analysis of the previous studies has revealed that wear-resistant and hard WN plays a crucial role in the high performance of the binary nitride multilayers [24,25]. However, the second layer is also undoubtedly significant. The effect of its composition on the ultimate properties of the coating is noteworthy and should be thoroughly investigated. It can enhance the functional properties of tungsten nitride or deteriorate them. Additionally, an interesting finding is that all WN-based binary nitride multilayers addressed in previous works were synthesized solely by magnetron sputtering and ion-beam-assisted deposition methods. The cathodic-arc physical vapor deposition (CA-PVD) has never been used to produce such coating systems. The output of this method is highly ionized which allows controlling the energy with which ions impinge on the substrate. Among advantages of the CA-PVD technique are its compatibility with industry, material evaporation under low voltage (10–15 V), good adhesion, high deposition rate, precise stoichiometric control, low temperature, as well as the formation of the compact and uniform multilayer coating [26]. Therefore, the main goal of the present study was to investigate the effect of the second layer on the microstructure and mechanical and tribological properties of CA-PVD WN-based multilayer coatings. Among various binary TMNs (transition metal nitrides), CrN, ZrN, MoN, and NbN were chosen as layers alternating with tungsten nitride. Chromium and zirconium nitrides have proven their positive effect on multilayers’ high-temperature performance and the ability to work in synergy with WN in one structure. Molybdenum nitride is expected to reduce the wear and coefficient of friction (COF) of the multilayer system by forming the lubricant Magnéli phase [4,27,28]. The introduction of NbN should optimize the performance of WN due to its superior hardness and wear resistance [29,30]. As far as the authors are aware, the combination of WN and MoN in one multilayer structure has not been studied yet.

In the present study, nanoscale WN/MeN (Me = Cr, Zr, Mo, Nb) multilayer coatings synthesized by CA-PVD at the same deposition parameters have been comprehensively investigated. The first-principles calculations of random W_1−y_N_1−x_ (0 ≤ x, y ≤ 1) phases are provided to better understand the most probable structure of the WN layers in the deposited multilayers. The aim was to study the effect of the second layer (nanolayer), deposited on top of the WN, on the phase composition, microstructure evolution, and performance characteristics of multilayers. Moreover, the comparison of the structural features, mechanical properties, wear resistance, friction performance, and adhesion strength are provided to choose the most suitable binary nitride for the WN-based multilayer. The formation of the nanocomposite structure in MoN and NbN layers growing on the cubic tungsten nitride has been investigated.

## 2. Materials and Methods

### 2.1. Deposition Procedure

The WN/MeN (Me = Cr, Zr, Mo, Nb) multilayer coatings were deposited via the CA-PVD using upgraded “BULAT-6” vacuum-arc equipment. The X6CrNiTi18-11 stainless steel (Kharkiv Institute of Physics and Technology, Kharkiv, Ukraine) was utilized as a substrate. Prior to the deposition, the substrates were mechanically ground and then polished on a polishing cloth with Goya’s paste. After that, they were ultrasonically cleaned in acetone, and then the residual surface contaminants were removed by Ar^+^ ion bombardment in the vacuum chamber. Different coating systems were prepared using five pure metal targets: W, Cr, Zr, Mo, and Nb (99.5% purity) (Kharkiv Institute of Physics and Technology, Kharkiv, Ukraine). During the deposition process, the working pressure was 0.73 Pa, and the substrate temperature was kept at 400 °C. The substrates were biased to −150 V, and the applied arc currents were 100 A. The target-substrate distances were fixed at 60 mm. For better adhesion, a thin interlayer at the substrate/coating interface was deposited for 1 min. It was a metal other than tungsten (Cr, Zr, Mo, or Nb), depending on the second layer. Hereafter, multilayer coatings were prepared in the nitrogen atmosphere by the alternating deposition of nanoscale WN and CrN, ZrN, MoN, or NbN layers. The total deposition time for each coating system was 60 min, and the rotation rate for all substrates was 7 rpm, resulting in coatings consisting of 420 layers.

### 2.2. Chemical Composition and Microstructure Analysis

The phase analysis was conducted by Bragg–Brentano X-ray diffraction (XRD, Panalytical Empyrean X-ray diffractometer, U = 40 kV and I = 40 mA) (Malvern Panalytical Ltd., Malvern, UK) with Cu-Kα radiation (λ = 1.5406 Å). An incident beam path consisted of a fixed programmable divergence slit (0.5°) with a fixed anti-scatter slit (1°), a nickel β filter, a Soller slit of 0.04 rad, and a 10 mm fixed mask. A diffracted beam path was fitted by a PIXcel3D-Medipix3 1 × 1 detector (Malvern Panalytical Ltd., Malvern, UK) in scanning line (1D) mode with an active length of 3.3482° 2θ and a number of active channels of 255, Soller slit of 0.04 rad, and a programmable anti-scatter slit of 0.5°. XRD patterns were collected within the 2θ ranging from 20° to 90° at 0.013° step size. Residual stresses were analyzed by the XRD technique using the same device. For the measurements of residual stresses, the ω-2θ method with Co-Kα radiation (λ = 1.78901 Å) at U = 40 kV and I = 30 mA was utilized. An incident beam path was fitted by a parallel beam X-ray mirror with a 10 mm fixed mask, fixed divergence slit of 0.5°, Soller slit of 0.04 rad, and fixed anti-scatter slit of 0.5°. The diffracted beam path comprised a PIXcel3D-Medipix3 1 x 1 detector in scanning line (1D) mode, Soller slit of 0.04 rad, an iron β filter, and a programmable anti-scatter slit of 0.5°. The (311) diffracting planes of NbN, MoN, CrN, and ZrN at 2θ = 80°, 80°, 88°, and 88°, respectively, were chosen to determine the residual stresses by the sin^2^ψ method. The measuring ranges were set from 65° to 95° 2θ for (311) NbN and (311) MoN and from 77° to 98° 2θ for (311) CrN and (311) ZrN, with a step size of 0.3°. Elastic moduli required for the residual stresses calculations were taken from the nanoindentation measurements. Poisson ratios were considered from the literature [31,32,33,34].

The morphology and cross-sections of the WN/MeN (Me = Cr, Zr, Mo, Nb) multilayers were studied via the JEOL JSM 7600F high-resolution field emission scanning electron microscope (FE-SEM, JEOL Ltd., Tokyo, Japan) in backscatter electron imaging (BSE). It was equipped with the Oxford Instruments Inca Wave spectrometer (Oxford Instruments, High Wycombe, UK) for wavelength-dispersive X-ray spectroscopy (WDS), used for the chemical composition characterization. Prior to the quantitative analysis of the coatings, the spectrometer was calibrated with Cr, Zr, Mo, Nb, W, and BN standards (Micro Analysis Consultants, St. Ives, UK). Kα (N and Cr), Lα (Zr, Nb, and Mo), and Mα (W) lines were selected for the quantification of X-ray data. The cross-sections were prepared by mechanical grinding and polishing of the samples. The total thickness of multilayer coatings was measured via calotest (Anton Paar Compact CAT^2^c Calotester, Anton Paar GmbH, Graz, Austria) at room temperature and humidity of about 60%. Auger analysis was carried out in a JEOL JAMP 9510-F field emission Auger microprobe (JEOL Ltd., Tokyo, Japan) at the energy of 10 keV and probe current of 10 nA with a tilt angle of 30° and 65° take-off angle. Auger spectra were evaluated in derivative form, and depth profiling was conducted via sputtering with 500 eV Ar^+^ ions to suppress the effect of atomic mixing. The topography and surface roughness were determined by the Zeiss LSM 700 scanning laser confocal microscope (LSCM) (Carl Zeiss Microscopy GmbH, Jena, Germany) following the ISO 25178 standard. The diode laser with a wavelength of λ = 405 nm was used as a radiation source. The total area of 166 × 169 μm^2^ was analyzed using an objective with a numerical aperture of 0.95 and 100× magnification. The 3D topography maps were stitched and constructed with Zeiss ConfoMap software (ConfoMap Premium 7.2, Digital Surf, Besançon, France).

The WN/NbN sample for the transmission electron microscope (TEM, JEOL Ltd., Tokyo, Japan) observations was prepared by focused Ga^+^ ion beam by Tescan Lyra 3 dual-beam scanning electron microscope (Tescan Orsay Holding, a.s., Brno-Kohoutovice, Czech Republic). The final thickness of the lamella was less than 50 nm. Milling was performed at the acceleration energy of 30 KeV. For minimization of surface damage, the energy of the gallium beam was decreased for fine polishing. The WN/MoN lamella was prepared using the Nanoanalytik Zeiss Auriga 60 high-resolution focused ion beam scanning electron microscope (FIB-SEM, Carl Zeiss Microscopy GmbH, Jena, Germany). First, a 20 × 2 × 2 µm carbon/platinum protective multilayer was deposited on the sample surface. Then trenches with a width of 1–2 µm around the sample position, forming a rectangle, were cut. They were made to decrease the influence of the intrinsic stresses of the layers on the lamella preparation in order to prevent its breaking. After that, the material on both sides of the protection layer was being removed with beams of 16 nA and 4 nA at 30 keV until the thickness of the lamella under the protection layer reached about 1µm. Then a lift-out and a mounting of the lamella onto a TEM copper grid were performed. At the final step, a thinning occurred with decreasing beam currents (from 600 pA to 50 pA at 30 kV), and final polishing with the beam of 240 pA at 5 kV took place.

The crystallographic characterization and phase identification of the WN/NbN and WN/MoN multilayers were carried out via transmission electron microscopy using JEOL JEM-ARM200CF double corrected field emission atomic resolution analytical transmission electron microscope (JEOL Ltd., Tokyo, Japan) working at 200 kV in bright-field (BF), high-resolution (HR-TEM), high angle annular dark-field (HAADF), and atomic resolution (AR-TEM) modes. The probe convergence semi-angle for all STEM measurements was set to 22 mrad and the inner-collection semi-angle of the HAADF detector was 90 mrad. Energy-dispersive X-ray spectroscopy (TEM-EDX) was performed using JEOL JED-2300 0.98 steradian solid-angle silicon drift detector (SDD, JEOL Ltd., Tokyo, Japan). The probe current 200 pA and 0.2 ms/pixel dwell time were used for TEM-EDX mapping of N, Nb, Mo, and W. Results were processed using Gatan DigitalMicrograph^®^ (GMS 3, Gatan, Inc., Pleasanton, CA, USA) and CrysTBox software (CrysTBox 1.10, Institute of Physics of the Czech Academy of Sciences, Praha, Czech Republic) [35].

### 2.3. Mechanical Properties and Tribological Behavior

Nanohardness and reduced Young’s modulus were evaluated using the Anton Paar NHT^2^ nanoindenter (Anton Paar GmbH, Graz, Austria) equipped with a Berkovich tip at the load of 10 mN. The dwell time was set to 5 s, while the loading and unloading rates were 40 mN/min. The load-displacement curves were processed by the Oliver and Pharr method [35]. In order to avoid the substrate contribution, indentation depths did not exceed 10% of the overall thickness. Nanoindentation measurements were performed at room temperature. The obtained results for each coating were the average values of 16 measurements (4 × 4 pattern). Standard deviations were represented as error bars. Scratch testing was carried out to determine the substrate/coating adhesion strength by Bruker UMT Tribolab device (Bruker Corporation, Billerica, MA, USA) adjusted to scratch test configuration with Rockwell-C type indenter. The load applied perpendicular to the surface increased linearly from 0 to 60 N at a rate of 10 N/mm. The sliding velocity and distance were 0.1 mm/s and 6 mm, respectively. The detailed examination of the first cracks and delaminations with a determination of critical loads was conducted by FE-SEM in secondary electron imaging (SEI). The adhesion of the WN/NbN coating system to the substrate was additionally examined by the Daimler–Benz test (Škoda RB-1 hardness tester, Škoda, Plzeň, Czech Republic). The Rockwell-C indentor was used to make imprints on the free surface of coatings at a load of 1500 N; the dwell time was set to 10 s. Furthermore, the character of cracks around imprints was evaluated with FE-SEM according to VDI 3198 standard.

The COF and wear resistance were studied via the ball-on-disk tests (Bruker UMT Tribolab Tribometer). The counter-body was an Al_2_O_3_ ball with a diameter of 5 mm. It was sliding against the coating during 1080 s at the normal load of 10 N and the velocity of 300 rpm. The total sliding distance was 85 m, and the radius of the wear track was 2.5 mm. All tests were conducted at room temperature and a humidity of 60%. The wear rate, *W*, was calculated using the following equation in accordance with the ASTM G99-17 standard:

W=V(Fp L),

where *V* is the volume loss, *F_p_* is the normal load, and *L* is a sliding distance. The unit of the specific wear rate is mm^3^/Nm. Wear volumes were measured by Zeiss LSM 700 LSCM.

### 2.4. Computational Aspects

To model the random B1-W_1−y_N_1−x_, 0 ≤ x, y ≤ 1, compounds, we considered the B1-W_16_N_16_ supercells. Since both the metal and non-metal sublattices are disordered, we used already published 16-atom special quasi-random structures (SQS) for FCC random alloys [36]. Thus, the SQSs used in the present study correspond to the W_1−y_N_1−x_ compositions with y = *n*/16 and x = *m*/16, where *n* and *m* are numbers of the vacancies in the W and N sublattices, respectively.

First-principles calculations were performed using the pseudopotential code “Quantum ESPRESSO” [37]. Vanderbilt ultra-soft pseudopotentials were used to describe the electron–ion interaction, and the generalized gradient approximation was employed to describe the exchange-correlation energy and potential [38]. The Gaussian smearing scheme with a smearing parameter of 0.272 eV was used for the Brillouin zone integration. The plane-wave cut-off energy was 516.8 eV. We used 15–20 special Monkhorst–Pack *k*-points depending on the SQS composition. The SQSs were optimized using the Broyden-Fletcher–Goldfarb–Shanno (BFGS) algorithm that allows the simultaneous relaxation of both cell vectors and atomic coordinates [38]. The relaxation was finished when the atomic forces were less than 25.7 meV/Å, the stresses were lower than 0.05 GPa, and the change of the total energy during the structural optimization was less than 1.36 meV.

The formation energy of W_1−y_N_1−x_ is

H^f^ = [E_T_(W_1−y_N_1−x_) − (1 − y)·E_T_(W) − 1/2·(1 − x)·E_T_(N_2_)]/(2 − x − y),

where E_T_(W_1−y_N_1−x_), E_T_(W), and E_T_(N_2_) are the total energies of W_1−y_N_1−x_, body-centered cubic tungsten, and molecule of solid N_2_, respectively.

The elastic moduli of W_1−y_N_1−x_ were investigated using the “ElaStic” code [39]. For polycrystalline random tungsten nitrides, the Hill bulk (B), shear (G), and Young (E) moduli were estimated using calculated elastic constants *c_ij_* [39]. The Vickers hardness (H_V_) was assessed using an empirical model H_V_ = 0.92·k^1.137^·G^0.708^, where k = G/B [40]. The phonon spectra were calculated using the PHONOPY code [41]. The PCW software [42] was used to calculate the XRD spectra.

## 3. Results and Discussion

### 3.1. Microstructure and Chemical Composition

**Figure 1:**
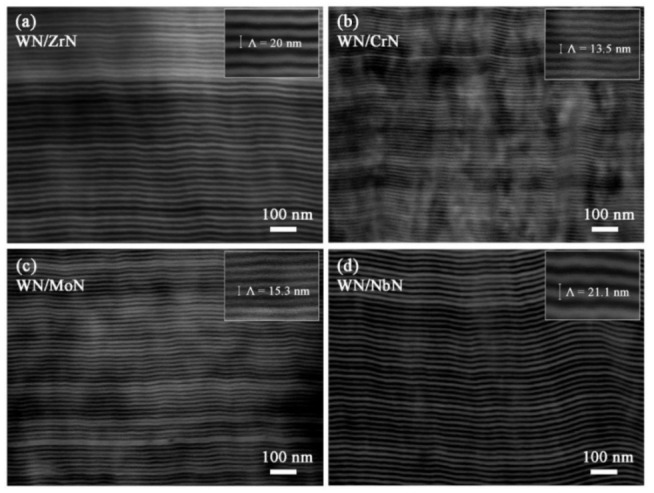
Cross-sectional SEM images of WN-based multilayer coating systems. Bilayer thicknesses are presented in the inserts.

The cross-sectional morphologies of the WN/ZrN, WN/CrN, WN/MoN, and WN/NbN coatings are presented in Figure 1. All multilayers demonstrated a well-defined layered structure with dense columnar growth. Bright layers were assigned to the WN, and dark ones belonged to the MeN (Me = Zr, Cr, Mo, Nb). In backscatter electron imaging, the heavier elements can deflect incident electrons stronger than light elements. Hence, heavier elements appear brighter. The inserts in Figure 1 show magnified SEM images with measured bilayer periods (Λ). Thus, coatings had different Λ: 14.5 nm (WN/CrN), 15.3 nm (WN/MoN), 20 nm (WN/ZrN), and 21.1 nm (WN/NbN). Respectively, the total coating thicknesses ranged from 4.7 μm to 5.6 μm. The decrease in bilayer thickness of multilayers deposited from Cr and Mo cathodes may be explained by the difference in kinetic energies of ions generated during the cathodic arc evaporation. Its distinctive feature is the formation of ions with high energies varying from 20 to 200 eV [43]. Among the five pure metal cathodes used for coating synthesis, Zr, Nb, and W produce ions with close energy values. However, the Cr and Mo ions significantly differ from the W ones (E_Cr_ = 71.6 eV, E_Mo_ = 149 eV, and E_W_ = 117 eV) [44]. That fact can cause resputtering and intermixing during the deposition and reduce the bilayer thickness. 

**Figure 2:**
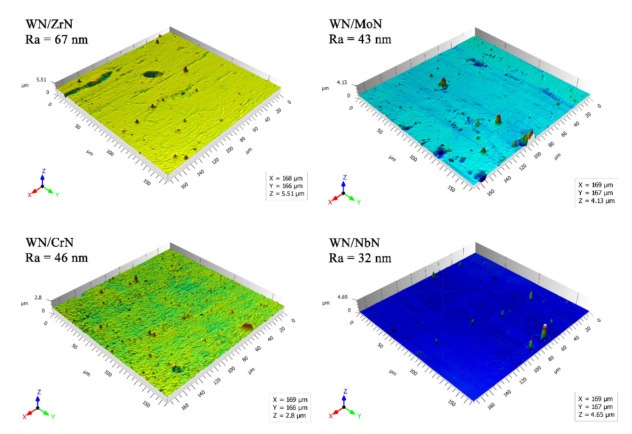
Surface topography and roughness of WN/ZrN, WN/CrN, WN/MoN, and WN/NbN multilayers.

The surface roughness is an essential parameter for protective coatings. It plays a decisive role in their wear behavior and friction performance. The irregularity of the surface is related to the pinholes and macroparticles ejected from the cathode spots [45]. Larger macroparticles are often found in coatings deposited from the cathode with a lower melting point [46,47,48]. Therefore, the refractory metals are expected to produce lower coating roughness. Deposited nanoscale WN-based multilayer systems confirmed this assumption: coatings containing Zr and Nb were the coarsest and the smoothest. The surface roughness of WN/ZrN, WN/CrN, WN/MoN, and WN/NbN was measured to be about 67 nm, 46 nm, 43 nm, and 32 nm, respectively (see Figure 2). All coatings had macroparticles of different sizes, which are typical for the cathodic arc evaporation process. The chemical composition of the deposited multilayers was determined by the WDS method (Table 1). It is accurate enough for detecting light elements such as N, has a low measurement error of about 1 at.%, and high spectral resolution [49]. Multilayer coatings had almost the same contents of W and N, except for the WN/CrN one. It contained a high tungsten concentration (37.3 at.%). Such peculiarity could be caused by the resputtering process due to significant differences in the kinetic energies and sizes of W and Cr ions. 

**Figure 3:**
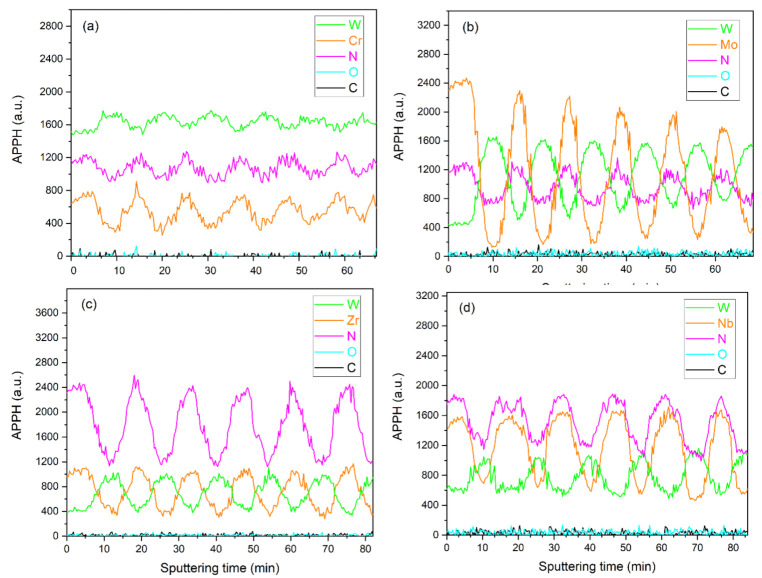
Auger in-depth profiles of the nanoscale multilayers: (**a**) WN/CrN, (**b**) WN/MoN, (**c**) WN/ZrN, and (**d**) WN/NbN.

Figure 3 shows the Auger in-depth element profiles of the multilayer WN-based coatings. The signals from the elements were clearly distinguished as a result of the qualitative analysis. The spectra demonstrated periodic oscillations of Auger electrons, which indicated the multilayer architecture of the coatings. An alternation of WN and MeN (Me = Zr, Cr, Mo, Nb) layers could be observed. It should be noted that the nitrogen profile has reached its minimum in the layer containing tungsten, which indirectly confirms the formation of the cubic W_2_N phase, in which N atoms occupy half of the octahedral sites. Furthermore, a decrease in the intensity of Auger signals from the coating elements was observed in the WN/CrN and WN/MoN profiles. This phenomenon was not associated with the effect of mixing elements between layers in the process of the sequential sputtering of the analyzed material. It was likely caused by the waviness of the near-surface regions of multilayers with CrN and MoN, which was observed during the detailed study of their cross-sections [50].

**Figure 4:**
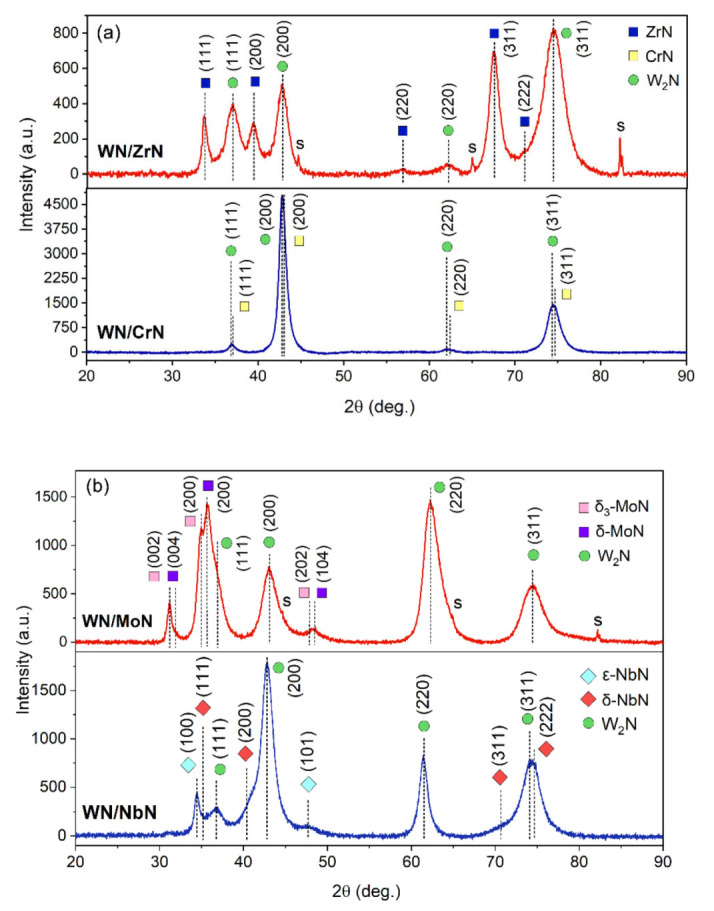
XRD patterns of the WN-based multilayer coatings: (**a**) WN/CrN and WN/ZrN; (**b**) WN/MoN and WN/NbN.

The XRD spectra of the nanostructured WN/MeN (Me = Zr, Cr, Mo, and Nb) coatings are presented in Figure 4. The phase analysis demonstrated that the common for all multilayers WN layer had the fcc NaCl-type W_2_N phase (JCPDF 00-025-1257). According to Deng et al. [51] and Polcar et al. [24,52], the single-phase fcc structure in tungsten nitride can be formed at 30 at.% < N < 55 at.%. Therefore, that is in good agreement with the findings of the present study. However, the second distinguishing nitride layer was unique for each coating system. Regarding the structure of the second layer, coatings could be roughly classified into two groups: (i) WN/ZrN and WN/CrN (Figure 4a), (ii) WN/MoN and WN/NbN (Figure 4b). 

The general trend inherent in the first group of multilayers was an isostructural growth during deposition. The XRD results showed that CrN and ZrN layers, similarly to WN, developed the NaCl-fcc Fm$\bar{3}$m structure. The spectra in Figure 4a exhibited (111), (200), (220), and (311) diffraction peaks of CrN (#225, Fm$\bar{3}$m, JCPDS 11-0065), ZrN (#225, Fm$\bar{3}$m, JCPDS 35-0753), and W_2_N (#225, Fm$\bar{3}$m, JCPDS 25-1257) phases. In the WN/ZrN coating, the (311) reflections were a bit more dominant, but no strong preferred orientation was observed. However, the WN/CrN had a noticeable (200) texture. Due to the similar lattice parameters (mismatch of 0.45%), the CrN and W_2_N peaks were overlapped in the XRD pattern. On the contrary, the phases constituting the WN/ZrN coating were clearly separated since the difference between fcc lattices was about 8.1%. Moreover, the diffraction peaks were shifted towards smaller angles. One of the reasons for that can be internal stresses, which occurred during the deposition process. Another explanation is compressive residual stress caused by the mismatch of the thermal expansion coefficients (CTE) of the stainless-steel substrate and coating and between different ceramic binary layers. The WN/CrN and WN/ZrN coatings developed compressive residual stresses of about −7462.7 ± 245.5 MPa and −4865.6 ± 306.6 MPa. The more minor peak shifts towards lower 2θ angles were observed in the (200) and (111) planes of ZrN and W_2_N, respectively. That could be caused by the effects of the stacking faults and lattice strain.

A distinctive feature of the multilayers included in the second group was a polycrystalline structure consisting of the fcc and hexagonal phases in MoN and NbN layers. However, the WN layer in both coating systems exhibited only fcc structure. It has been found that the WN_x_ coatings undergo a transformation from the bcc-W (N < 0.08) to the mixed structure of bcc-W and fcc-W_2_N (N > 0.12), then to the single-phase fcc-W_2_N (N > 0.32) and eventually to the single-phase hcp-WN (N > 0.55) [25]. Thus, the nitrogen pressure chosen in the present paper was enough to form the single-phase fcc-W_2_N in all coatings. The phase composition variations (cubic, hexagonal, tetragonal) are mainly characteristic of the transition metal nitrides of groups V and VI [53]. 

The WN/MoN coating consisted of the fcc-W_2_N, hexagonal δ-MoN (# 162, P$\bar{3}$1m) [54] and δ_3_-MoN (#186, P6_3_mc) [28], and fcc-Mo_2_N (#225, Fm$\bar{3}$m) [55,56] phases (Figure 4b). XRD did not detect the cubic γ-Mo_2_N, but a more detailed HR-TEM study proved its presence. High nitrogen pressure means a high number of nitrogen atoms. According to the WDS analysis, the N content was about 54.2 at.%, which contributed more to the formation of the stoichiometric hexagonal MoN phases [47]. All peaks were shifted to the lower diffraction angles, indicating the increase in the lattice parameters of δ_3_-MoN and δ-MoN phases to the following values: a = 5.91 Å, c = 5.729 Å and a = 5.787 Å, c = 5.622 Å, respectively. The excess nitrogen in the lattices results in their expansion, which leads to higher compressive stresses [4]. The coating had compressive residual stress of about −7226.2 ± 618.8 MPa.

The WN/NbN coating developed the fcc W_2_N, fcc δ-NbN (#225, Fm$\bar{3}$m, JCPDS 74-1218), and hexagonal ε-NbN (#194, P6_3_/mmc, JCPDS 89-4757) phases [57]. The NbN layer exhibited a nanocomposite structure, as was observed in Ref. [30]. A nanocomposite is a multiphase material in which one of the phases has one, two, or three dimensions of less than 100 nm [58]. For instance, the material can consist of a combination of nanograins with different crystallographic orientations and/or different phases [8]. According to the study of these crystal structures, the nearest Nb atoms are placed closer to each other in ε-NbN compared to the NaCl-type NbN, and the hexagonal lattice has been found to be more stable [59,60]. The deposition parameters favored the formation of the W_2_N phase with (200) texture, ε-NbN with (100), and δ-NbN with (200) preferred orientations. Li et al. [30] studied NbN coatings with the same nanocomposite structure, demonstrating enhanced toughness and hardness. According to the XRD pattern of WN/NbN, lattice parameters of the W_2_N and ε-NbN phases were increased to 4.22 Å and a = 3.005 Å, c = 11.3 Å, respectively. Meanwhile, the cubic δ-NbN lattice decreased to a value of 4.344 Å. The latter manifested in a slight shift of diffraction peaks towards higher angles. Such changes in the peak positions can be caused by the thermal stresses and residual strain after the ion bombardment during the deposition [47]. According to the sin^2^ψ measurements, the NbN coating had compressive residual stresses of about −9235.6 ± 233.1 MPa.

**Figure 5:**
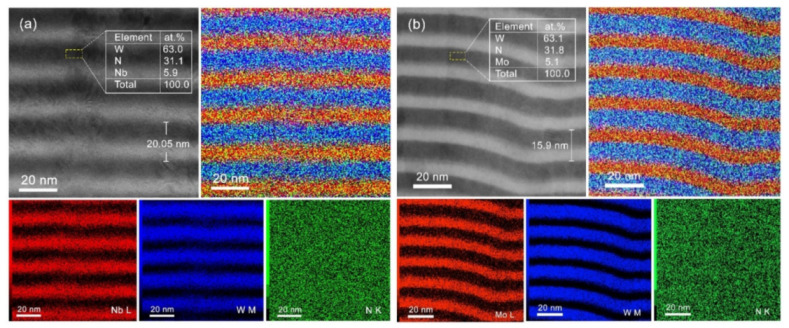
Cross-sectional BF STEM images of (**a**) WN/NbN and (**b**) WN/MoN multilayer coatings with element concentrations in WN layers. STEM-EDX maps of Nb L/Mo L, W M, and N K with the superposition X-ray maps.

The cross-sectional bright-field STEM images of WN/NbN and WN/MoN coatings are presented in Figure 5. It was found that the bilayer thicknesses of the multilayers with Nb and Mo-containing layers were 20.05 nm and 15.9 nm, respectively, which was in good agreement with SEM observations. The STEM-EDX mapping of the W, Nb/Mo, and N elements demonstrate multilayer architecture with clearly seen alternating WN and NbN or MoN layers. The EDX results for the WN layer of both coatings showed that the W/N ratio was approximately 2, proving the formation of the cubic W_2_N phase. About 5.9 at.% of Nb was also detected, originating from the neighboring layers.

**Figure 6:**
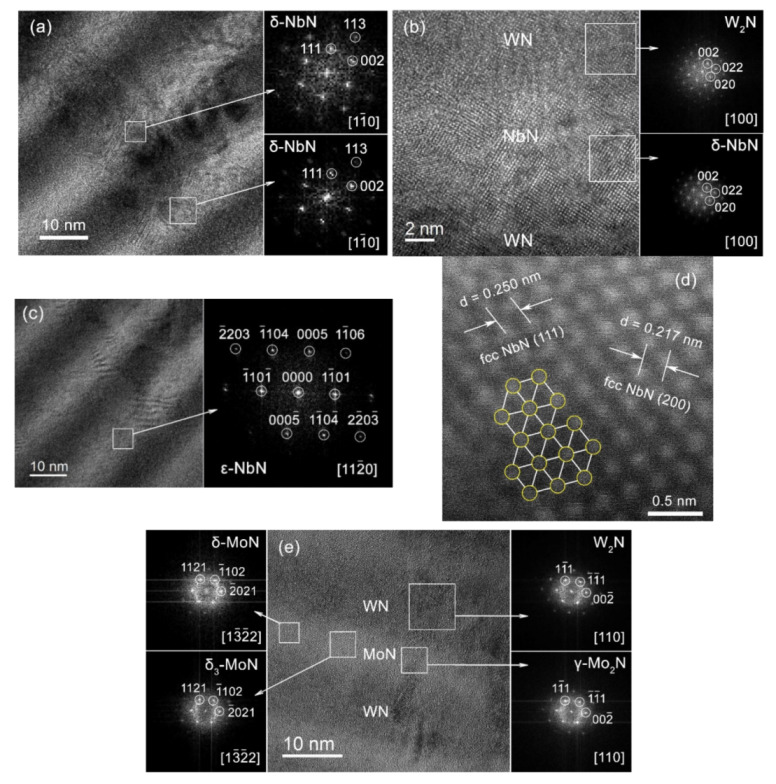

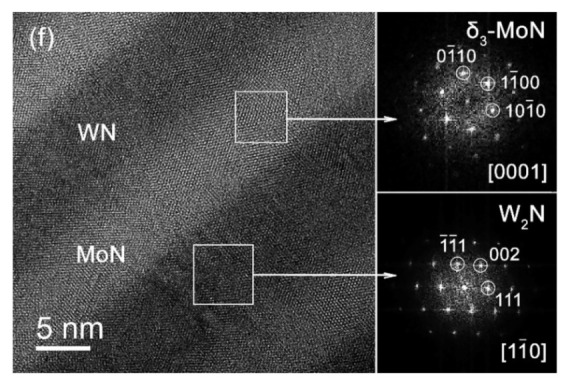
Cross-sectional HR-TEM images of the WN/NbN coating with relevant FFT patterns from different layers corresponding to (**a**) [1$\bar{1}$0] zone axis of the cubic δ-NbN, (**b**) [100] zone axis of W_2_N and δ-NbN phases, and (**c**) [11$\bar{2}$0] zone axis of hexagonal ε-NbN. (**d**) HAADF STEM image of the columns of Nb atoms in fcc δ-NbN lattice oriented along <110> direction. HR-TEM cross-sections of the WN/MoN multilayers with the corresponding FFT patterns featuring (**e**) hexagonal δ-MoN and δ_3_-MoN, and cubic γ-Mo_2_N and W_2_N phases; (**f**) [0001] zone axis of δ_3_-MoN phase and [1$\bar{1}$0] zone axis of the fcc W_2_N.

Figure 6 presents the cross-sectional HR-TEM and HAADF STEM images of the WN/NbN multilayer. From the point of crystallography, the estimation of fast Fourier transform (FFT)-HR-TEM of the WN layer (Figure 6b) revealed the crystallite of cubic (fcc) W_2_N phase, oriented along the [100] zone axis. In contrast, the NbN layers consisted of both hexagonal ε-NbN and fcc δ-NbN phases. Figure 6a,b present the crystallites of fcc δ-NbN oriented along [1$\bar{1}$0] and [100] zone axes and Figure 6c shows the FFT pattern of the hexagonal ε-NbN phase, oriented along [11$\bar{2}$0] zone axis. In both WN and NbN layers, the fcc crystallites exhibited two types of zone axes: <110> and <100>, however, due to the selected <110> orientation of TEM lamella, <110> NbN and <110 >W_2_N crystallites were predominant. Figure 6d demonstrates the more detailed atomic resolution HAADF STEM image recorded from fcc δ-NbN phase. The image shows columns of Nb atoms arranged in pseudo-hexagons, which is in agreement with <110>-oriented δ-NbN. The measured interplanar distances of fcc-NbN were the following: d_200_ = 0.217 nm and d_111_ = 0.250 nm, which is consistent with the XRD analysis.

The typical microstructure of the CA-PVD WN/MoN multilayers studied by HR-TEM is presented in Figure 6e,f. The individual alternating layers can be clearly distinguished. Analysis of the FFT patterns proved the XRD findings. The WN layer consisted of a cubic W_2_N phase similarly to the WN/NbN sample. However, the MoN layer developed a nanocomposite structure, represented by three crystalline phases: fcc γ-Mo_2_N and two hexagonal phases, δ-MoN and δ_3_-MoN. The cubic molybdenum nitride was not detected in XRD patterns because of the small amount.

### 3.2. First-Principles Calculations of Cubic W_2_N

Above, we assumed that, in the WN/MeN (Me = Zr, Cr, Mo, Nb) coatings, the WN layers were composed of the cubic W_2_N (WN_0.5_) phase. Considering that substoichiometric tungsten nitrides were not yet studied theoretically, we performed first-principles calculations of the cubic random W_1−y_N_1−x_, 0 ≤ x, y ≤ 1, phases to study their stability and structural and mechanical properties. The results obtained could give more insight into the formation of these random phases and would enable us to establish the possible structure and composition of the WN layers in the deposited multilayers. 

**Figure 7:**
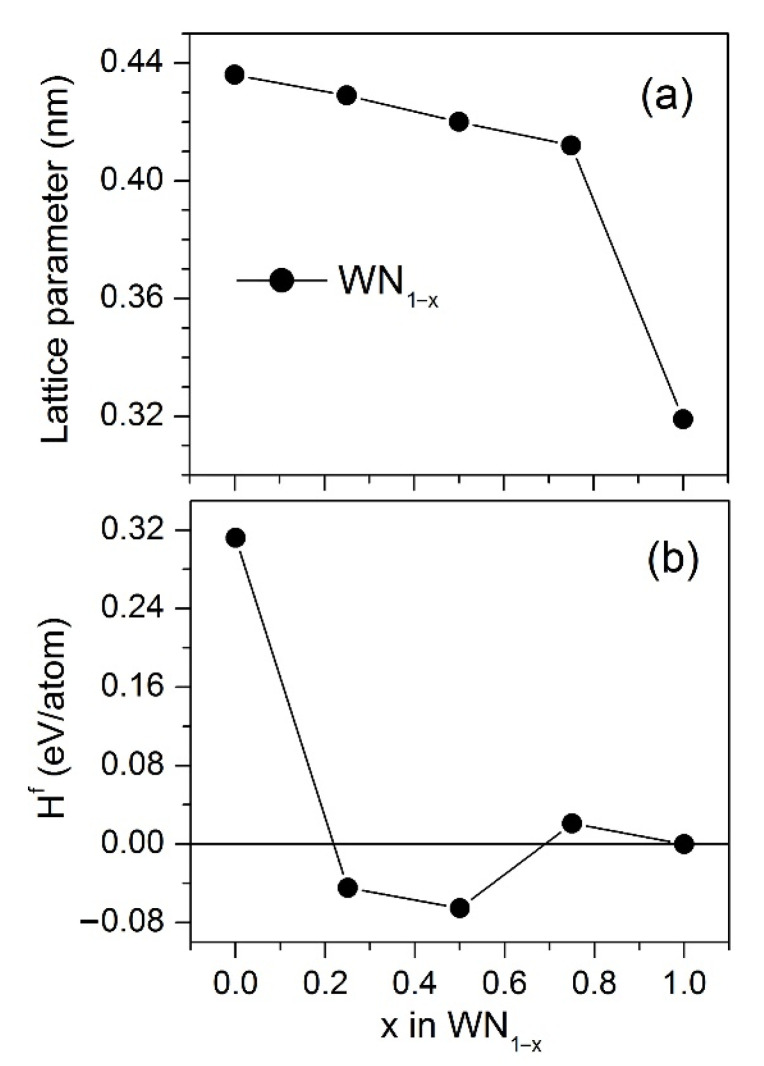
(**a**) Lattice parameters and (**b**) energy of formations, H_f_, of the random structures based on the W_16_N_16_ supercells, W_1−y_N_1−x_, with y = n/16 and x = m/16, where n and m are numbers of the vacancies in the W and N sublattices, respectively.

Figure 7 shows the lattice parameter and formation energy of the cubic random W_1−y_N_1−x_ phases as composition functions. For WN_1−x_, the lattice parameter decreases with increasing x, whereas the E^f^(x) dependence has a minimum at x = 0.5. The stoichiometric phase cannot be synthesized because its formation energy H^f^ = 0.3121 eV/atom is positive. For comparison, this value agrees well with the formation energy of 0.3115 eV/atom for WN obtained in Ref. [61]. For WN_0.5_, the calculated lattice parameter was about 0.420 nm. The experimental values of the lattice parameter of NaCl-type tungsten nitride in the present study were close to that theoretically predicted: 0.4224 nm for WN/ZrN, 0.4217 nm for WN/CrN, 0.419 nm for WN/MoN, and 0.4217 nm for WN/NbN. The WN_0.5_ phase is seen to be the most stable among other W_1−y_N_1−x_ compounds. This prompts us to suppose that the WN layers in the deposited nanolayered coatings are composed of the random cubic WN_1−x_ structures with compositions close to WN_0.5_. In such structures, the tungsten atoms form the fcc lattice, and the N atoms are randomly distributed at the octahedral sites. This conclusion is consistent with the results of the experimental investigations of substoichiometric tungsten nitrides [62].

**Figure 8:**
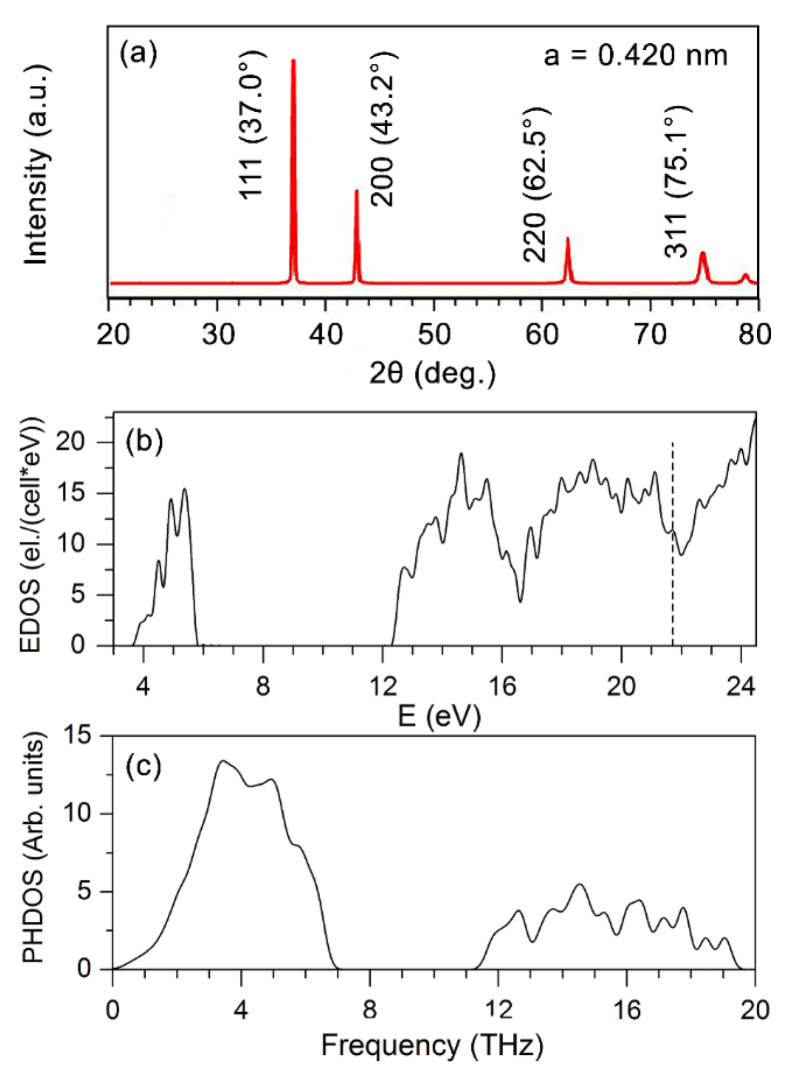
(**a**) Calculated XRD pattern for cubic NaCl-type WN_0.5_. (**b**) Electronic (EDOS) and (**c**) phonon (PHDOS) densities of states of WN_0.5_. The vertical line denotes the Fermi level.

We calculated the XRD spectrum for WN_0.5_ (Figure 8a) and compared it with the corresponding spectrum obtained experimentally in Ref. [63]. The patterns agreed rather well, indicating that our approach correctly reproduces the structure of the random WN_0.5_ phase. Additionally, the peak positions in the calculated spectrum are close to those in the XRD spectra of the deposited multilayers (Figure 4), which validates our conclusion about the structure and composition of the WN layers in them. 

We checked the elastic stability of WN_0.5_. A cubic crystal has only three independent constants: C_11_, C_12,_ and C_44_. For the random WN_0.5_ structure, there are 21 independent elastic constants. Since the structure of WN_0.5_ is very close to the cubic one, we adopt C_11_ = 1/3(c_11_ + c_22_ + c_33_), C_12_ = 1/3(c_12_ + c_13_ + c_23_), and C_44_ = 1/3(c_44_ + c_55_ + c_66_), where c_ij_ are the elastic constant of the disordered WN_0.5_ phase. The necessary and sufficient conditions for the elastic stability of a cubic crystal are C_11_ − C_12_ > 0, C_11_ + 2C_12_ > 0, C_44_ > 0. The values of C_11_, C_12_, and C_44_ were found to be 510.2, 236.1, and 122.6 GPa, respectively, and they clearly indicate that WN_0.5_ should be elastically stable. Figure 8b,c show the calculated electronic (EDOS) (a) and phonon (PHDOS) (b) densities of states of the WN_0.5_ phase. One can see that this phase will exhibit the properties inherent to metals, and the PHDOS points indicate that the WN_0.5_ phase should be elastically stable since there are no imaginary (negative) frequencies in its phonon spectrum. 

Given the calculated Hill elastic moduli B = 327.1 GPa, G = 129.1 GPa, and E = 342.3 GPa, we estimated the Pugh’s ratio k = G/B and Vickers hardness of WN_0.5._ The calculated Pugh’s ratio of 0.39 and the Poisson’s ratio σ = 0.33 suggest that WN_0.5_ should be ductile according to the criterion for ductility of a material: k < 0.6 and σ > 0.25 [64]. The hardness of 1000 HV (10 GPa) was found to be lower than 2600 HV (26 GPa), theoretically predicted for the NbO type WN in Ref. [61].

### 3.3. Mechanical Properties

**Figure 9:**
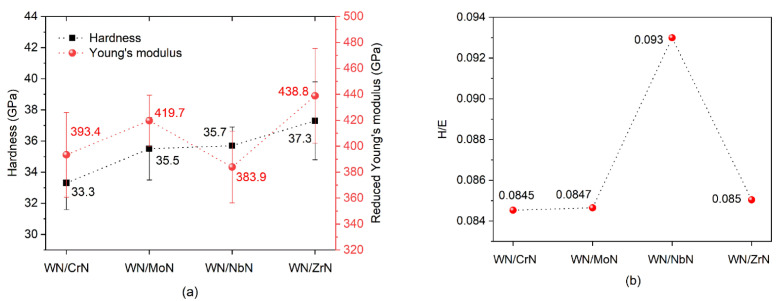
(**a**) Hardness and Young’s modulus, (**b**) H/E ratio of the WN-based multilayer coating systems.

The nanohardness (H), Young’s modulus (E), and H/E ratio (elastic strain to failure) are shown in Figure 9. The hardness of the fcc-W_2_N was reported to be around 24 GPa [14]. Therefore, all WN/MeN (Me = Zr, Cr, Mo, Nb) coating systems demonstrated enhanced hardness ranging from 33.3 ± 1.7 GPa to 37.3 ± 2.4 GPa. Such behavior can be explained by the mixed character of bondings in the constituent transition metal nitrides, [53]. Three bonds in different proportions occur in such binary nitrides: metallic (Me-Me), ionic (caused by the charge transfer between Me and N), and covalent (Me-N) components [65]. These bond contributions mainly determine the coating properties. For instance, the high hardness is attributed to the strong covalent component. However, materials with predominant covalent bonding are found to be brittle. That results in fast cracking, spalling, and degradation. Therefore, the transition metal nitrides with the optimal proportion between covalent and metallic components are expected to have enhanced tribomechanical properties. Moreover, the hardness of the WN-based multilayer coatings superior to the reported respective single-layer coatings can also be related to the multilayer architecture. Nanoscale layers demonstrate an increase in the grain boundaries’ volume fraction, which refers to the Hall–Petch strengthening. Besides, the alternation of layers with different properties provides the formation of numerous interfaces between layers hindering dislocation motions and crack propagations [50,66]. Hardening arises due to coherency strains in the presence of crystalline interlayers and due to shear modulus differences between the different phases, i.e., Koehler hardening [67,68].

Among the four WN-based multilayers, the WN/NbN demonstrated the best mechanical properties: high hardness (35.7 ± 1.2 GPa) combined with the lowest Young’s modulus (383.9 ± 27.6 GPa) (see Figure 9a). Young’s modulus is an essential property of the material that characterizes its stiffness. Whereas enhanced hardness is desirable for high-performance coatings used for tribological applications, Young’s modulus is supposed to be sufficiently low to improve resistance to deformation. Hence, coatings with a low Young’s modulus and a high H/E ratio of about 0.1 are considered elastic ones. These conditions were met in the case of the WN/NbN multilayer that manifested in a high H/E ratio value of 0.093 (see Figure 9b). Thus, this coating system is expected to have enhanced resistance to cracking and superior wear behavior. The described findings may be explained by the effect of the nanocomposite nature of the NbN layers consisting of two nanocrystalline phases (hexagonal and cubic) [30,66]. The δ’-NbN phase has a higher covalency level compared to the cubic one. It has been found that due to the covalent character, the hexagonal structure possesses enhanced hardness, which can explain the high hardness of the WN/NbN coating studied in the present paper [69]. It has been discovered that calculating the Cauchy pressure (c_12_–c_44_) for a certain material can provide information about its toughness. The negative values indicate the predominance of the directional bonding resulting in a more brittle character of the material. Conversely, positive values imply that it is more ductile [70]. The Cauchy pressure for δ-NbN and δ’-NbN phases was positive, meaning that they should be tough and ductile. However, the cubic structure with a higher number of dislocation sliding systems demonstrated superior to hexagonal NbN ductility [30]. Thus, a nanocomposite structure consisting of these two phases within the NbN layers and the multilayer architecture of WN/NbN coatings endows them with improved elasticity and cracking resistance.

### 3.4. Adhesion Strength

**Figure 10:**
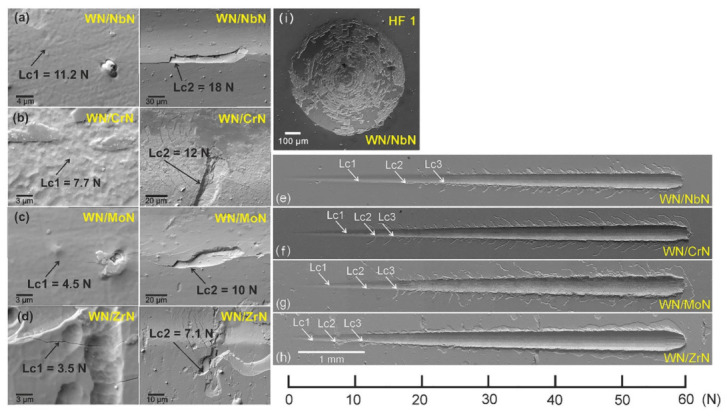
The adhesion strength of the WN/MeN (Me = Cr, Zr, Mo, Nb) multilayers to the stainless-steel substrate: (**a**–**d**) (from left to right) detailed SEM images of the first crack and first delamination; (**e**–**h**) general view of the scratches; (**i**) SEM image of the intent in WN/NbN sample after Daimler–Benz adhesion test.

Figure 10 presents the adhesion strength of the WN-based multilayer coatings to the stainless-steel substrate. The scratch test is a widely used method to study coating adhesion, a crucial parameter for tribological applications. Three critical loads (Lc_1_, Lc_2_, Lc_3_) were measured to evaluate the adhesion strength. They should be interpreted as follows: Lc_1_ load corresponds to the first crack initiation, Lc_2_ determines the first delamination of the coating, and Lc_3_ indicates the coating failure. The surface roughness, mechanical strength, and substrate material directly influence the critical load values [47]. Zhang et al. [53] demonstrated that hard CrN synthesized on soft 316 L stainless steel demonstrated the lowest adhesion strength compared to harder high-speed steel and cemented carbide substrates due to the continuous plastic deformation of the substrate. In the present study, we observed similar results for hard WN-based multilayer coatings due to the effect of a much softer substrate.

The detailed investigation of the first crack (Lc_1_) and delamination (Lc_2_) initiations in the WN-based multilayers correlated well with the above-discussed findings (Figure 10, left part). According to the scratch test results, the total failure of the WN/ZrN, WN/CrN, WN/MoN, and WN/NbN coatings were recorded at the following L_3_ values: 11.4 N, 15.0 N, 15.1 N, and 24.2 N, respectively. Hence, the WN/NbN multilayer demonstrated the best adhesion to the substrate, which was expected based on high mechanical properties and low surface roughness. Similarly, the WN/ZrN coating with the highest roughness showed the worst results. It has been found that Cauchy pressure that describes the angular characteristics of chemical bonds had negative values for ZrN, which indicates brittle behavior. Moreover, the coating failure could be caused by the difference in Young’s modulus values of the stainless-steel substrate and coating. The hard multilayer deposited on material with low modulus can easily chip and degrade when high loads are applied [53]. Thus, the WN/ZrN coating with the highest Young’s modulus developed numerous delaminations around the wide scratch groove and was accumulated at the edges, implying its adhesive failure (see Figure 10d). Other multilayer systems demonstrated only cracks around scratches. It should be noticed that coating with NbN was not aggregated along the scratch groove and resisted the increasing load even after its exfoliation. It showed the narrowest scar and the shortest peripheral cracks.

The Daimler–Benz test was additionally conducted to study the WN/NbN multilayer that demonstrated the best adhesion to the substrate during scratch testing. Figure 10e shows the SEM image of the imprint after the Rockwell-C indenter. The results revealed no evidence of the radial cracks and delaminations near the indentation, classifying the coating adhesion as HF 1 that refers to the best adhesion. Therefore, the soft steel substrate vastly influenced the adhesion strength during the scratch test. However, a detailed analysis of the WN/NbN multilayer allowed us to conclude that it had high adhesion suitable for tribological applications. It is recommendable to use high loads only for coatings deposited on hard substrates such as cemented carbide or high-speed steel.

### 3.5. Wear Performance

The wear behavior of the protective hard coatings can be assessed by two main parameters: friction coefficient and specific wear rate. They are responses of the tribosystem consisting of two materials sliding against each other [71]. The wear performance is mainly affected by the coating characteristics, surface asperities, counter-body material, media, applied load, and velocity. The wear-resistant coatings operating at dry-sliding mode are expected to possess low COF and specific wear rates to withstand plastic deformation. 

**Figure 11:**
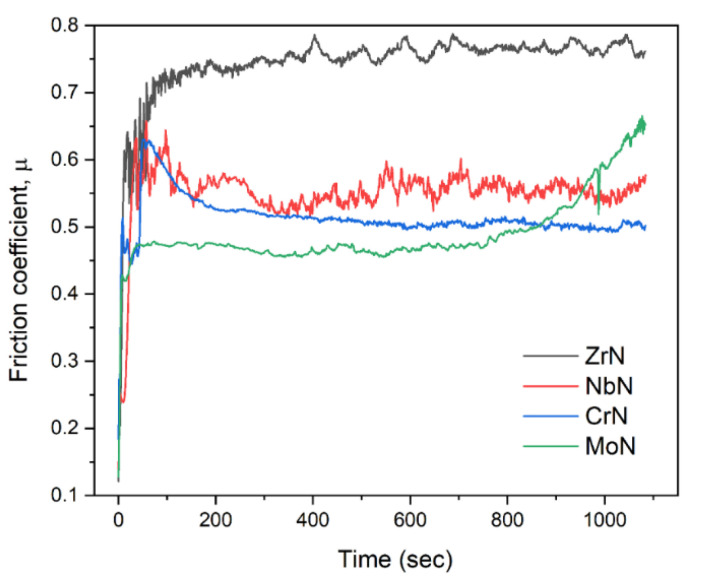
The friction coefficient curves of the WN/ZrN, WN/CrN, WN/MoN, and WN/NbN coatings on the stainless-steel substrate.

Figure 11 shows the coefficients of friction of the WN/MeN (Me = Zr, Cr, Mo, Nb) coatings. They were measured during the sliding of the Al_2_O_3_ ball against their surfaces. The two parts of the COF curves could be distinguished—running-in (fluctuating part at the beginning) and steady-state (stable part) periods. The COF of the WN/MoN and WN/NbN coatings within the first 50 s of rubbing reached steady-state values of about 0.47 and 0.55. The WN/ZrN coating showed a gradually increasing friction coefficient in a range of 0.74 to 0.78 after 180 s of the running-in period. The COF curve of the WN/CrN multilayer at 80 s abruptly reached the maximum (0.63) and then stabilized at 0.52. Among four WN-based multilayer systems, WN/ZrN had the worst friction performance. The explanation for this behavior may be the brittle nature of ZrN due to the prevalence of directional (covalent) bonding. It was manifested in the highest hardness of WN/ZrN among all deposited coating (37.3 ± 2.4 GPa). Furthermore, it had the highest roughness, which vastly deteriorates wear and friction behavior. Considering these facts, we can assume that repeated rubbing of alumina ball led to the detachment of asperities protruding from the coating surface with a further generation of hard wear particles due to cracking and brittle fracture, which could cause severe plastic deformation and plowing effect.

Even though WN/MoN multilayer had similar to WN/CrN mechanical properties and surface roughness, it exhibited the lowest COF among all multilayer coatings. The reason for that was the low friction coefficient of molybdenum nitride (~0.4). It forms lubricant Mo_n_O_3n−1_ oxides during friction, which are oxygen-deficient Magnéli-phases [4]. However, the friction coefficient of WN/MoN coating after 780 s of sliding began to rise to 0.66. That could mean that the multilayer lost its integrity and gradually was degraded. Wan et al. [72] observed the same behavior for MoN/TiSiN coating, which had a short running-in period and low steady value of COF, but after 600 s of testing, it started to increase due to the delamination. The friction coefficient curve of the WN/NbN multilayer system demonstrated a stable steady-state period till the end of the sliding. The rapid increase in the COF at the beginning was due to the grinding of surface asperities. After running-in state, the fluctuations could be caused by the repeated formation and removal of niobium oxide (Nb_2_O_5_) tribofilms, effectively resisting the shear load. It has been found that Nb_2_O_5_ can reduce COF even with enhancing a sliding velocity, has a low wear rate, and melts only at 1510 °C, while WO_3_ is stable only up to 800 °C [73,74]. Thus, the formation of the Nb_2_O_5_ and WO_3_ during the alumina ball sliding was expected to contribute to high wear resistance.

**Figure 12:**
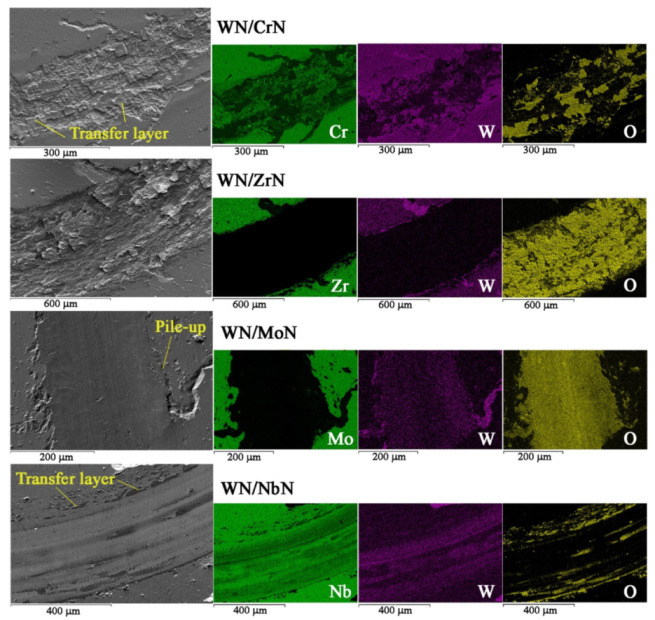
SEM images of wear scars (large images on the left) with EDS elemental distribution maps (smaller images on the right) of the nanocomposite WN/MeN (Me = Zr, Cr, Mo, Nb) multilayer coatings.

The typical wear tracks of WN/MeN (Me = Zr, Cr, Mo, Nb) coatings after the ball-on-disk test are shown in Figure 12. The specific wear rates of multilayers and corresponding counter-body Al_2_O_3_ balls were calculated to evaluate wear behavior. Table 2 summarizes the values of average COF and wear rates. The analysis of the wear scar features of all multilayers revealed that WN/NbN and ZrN coatings exhibited the best and the worst wear resistance, respectively.

SEM image of the WN/NbN wear track showed that the coating remained intact after alumina ball sliding (see Figure 12). The layers of WN and NbN were detected on the EDS maps, whereas no signs of Fe, the main element of the substrate, were found. The presence of oxygen contamination could be related to transferring the removed counter-body material and the formation of Nb_2_O_5_ and WO_3_ tribofilms appearing as islands. As it was mentioned before, those oxides protect the sliding surface of the coating against distribution. Moreover, low roughness, high elastic strain to failure, more ductile than brittle behavior of NbN, and mixing of hard nanocrystalline δ’-NbN and δ-NbN phases contributed to the superior wear performance. The calculated specific wear rates of the WN/NbN coating and counter-body were approximately 1.7 × 10^−6^ mm^3^/Nm and 2.0 × 10^−11^ mm^3^/Nm (Table 2). Accordingly, the Al_2_O_3_ ball was worn the most against the NbN-containing multilayer, while the latter exhibited the lowest wear rate among all coating systems. Hence, the wear mechanisms could be attributed to the mild oxidative wear. This regime belongs to the “tribochemical wear” group characterized by the activation of chemical reactions at the matting contact surfaces [71]. These reactions are possible due to the oxygen in the surrounding medium and “triboemission” (emission of exoelectrons, protons, and ions at the heated local parts) at the contact area. Thus, the nanoscale WN/NbN coating demonstrated the best wear resistance.

The WN/MoN coating had a higher wear rate of 8.6 × 10^−6^ mm^3^/Nm. The worn surface image displays the smooth wear scar with wear debris and material pile-up around the track edges. Some tungsten content still can be seen in Figure 12, but it was mainly in the form of oxides. Such wear scar of the WN/MoN multilayer indicates that lubricant Mo_n_O_3n−1_ phases participated in the coating protection from abrasive destruction. Such oxides can deteriorate the adhesion of the abrasive grain to the coating material [75]. The observed plastic deformation of the contact area was attributed to the flow wear mode.

The CrN-containing multilayer system after the ball-on-disk test partially lost its integrity. There was still tungsten detected, but the abrasive wear of the surface was clearly seen. The wear scar also contained chromium, but it was mainly ascribed to Cr in stainless-steel substrate. The wear debris transferred from the mating alumina ball was attached to the surface. That describes the wear mode as mild abrasive/adhesive. The WN/CrN coating exhibited the second-lowest specific wear rate of approximately 1.1 × 10^−5^ mm^3^/Nm with relatively low counter-body wear. 

According to the WN/ZrN multilayer EDS elemental mapping, the rough flake-like surface was observed after the alumina ball sliding (Figure 12). This multilayer and the corresponding counter-body were worn the most: their specific wear rates were 3.8 × 10^−4^ mm^3^/Nm and 5.9 × 10^−11^ mm^3^/Nm, respectively. As-deposited WN/ZrN coatings exhibited the highest surface roughness of about 67 nm. Due to numerous growth defects and brittle character of hard ZrN-containing multilayer, severe abrasive wear was the principal wear mode. The abrasive wear in brittle materials is manifested in easy crack formation because of the stress concentration in the sliding area with further flaky hard wear particle generation [71,75]. Those particles of various sizes have a plowing effect on the coating surface, leading to severe damage. 

Hence, high hardness is an important parameter expected to be inherent in the protective transition metal nitride coatings for tribological applications. However, it is hard to predict the wear behavior based solely on it. High elastic strain to failure (~0.1), which is defined as the ratio of hardness to Young’s modulus, has been found to be even more significant for wear resistance. The formation of the nanocomposite structure of the NbN layer, ductile nature of the δ’-NbN and δ-NbN phases, and high hardness and H/E ratio endowed WN/NbN with superior wear resistance. Therefore, among the four CA-PVD WN/MeN (Me = Zr, Cr, Mo, Nb) coating systems synthesized at the same deposition parameters, the WN exhibited the best characteristics when combined with NbN in a nanoscale multilayer architecture. The WN/NbN could be a perfect candidate for wear-resistant prospective coatings for tribological applications with extreme operating conditions.

## 4. Conclusions

The nanocomposite WN/MeN (Me = Cr, Zr, Mo, Nb) multilayer coatings deposited by CA-PVD have been comprehensively investigated. The effect of four different binary transition metal nitride layers on the microstructure, mechanical properties, friction performance, and wear behavior of WN-based multilayers can be summarized as follows:

(1) Coatings could be roughly divided into two groups according to their microstructure: (i) all constituent layers had NaCl-type cubic structure (WN/Zr and WN/CrN), and (ii) WN layers consisted of the fcc W_2_N phase, while other layers developed a combination of hexagonal and fcc NaCl-type cubic phases (δ-MoN, δ_3_-MoN, and γ-Mo_2_N in WN/MoN, as well as ε-NbN and δ-NbN in WN/NbN).
(2) To substantiate the formation of the substoichiometric tungsten nitride layers in the deposited multilayers and to gain more insight into their properties, first-principles investigations of the stability, structure, electronic, vibrational, and mechanical properties of the cubic random W_1−y_N_1−x_, 0 ≤ x, y ≤ 1, phases were carried out. The results show that the tungsten nitride layers in the deposited coatings can be composed of disordered cubic WN_1−x_ structures with compositions close to WN_0.5_. The WN_0.5_ phase is elastically and dynamically stable and exhibits the properties inherent to ductile materials.
(3) All WN/ZrN, WN/CrN, WN/MoN, and WN/NbN coating systems exhibited a high hardness range from 33.3 ± 1.7 GPa to 37.3 ± 2.4 GPa. The maximum elastic strain to failure (H/E ratio) value of about 0.093 was observed for WN/NbN multilayer. All coatings had similar friction coefficient values ranging from 0.47 to 0.55.
(4) The nanoscale WN/NbN multilayer coating showed the highest adhesion and had superior wear performance, remaining intact after the wear tests. It exhibited the lowest specific wear rate of about 1.7 × 10^−6^ mm^3^/Nm. That could be related to a low roughness, high elastic strain to failure, more ductile behavior of NbN, the nanocomposite structure (δ’-NbN and δ-NbN phases), and the formation of Nb_2_O_5_ and WO_3_ tribofilms during sliding. The WN/ZrN coating showed the worst wear resistance.

Therefore, the WN/NbN multilayer with the best wear resistance and mechanical properties holds promise to be effective as a hard protective coating for severe tribological applications.

## Figures and Tables

**Table 1:** Chemical composition of the WN/MeN (Me = Zr, Cr, Mo, Nb) multilayers.

Coating	Element Concentration (at.%)					
	W	N	Cr	Mo	Nb	Zr
WN/CrN	37.3	51.4	11.3	-		-
WN/MoN	26.4	54.2	-	19.4	-	-
WN/NbN	27.2	52.4	-	-	20.4	-
WN/ZrN	26.4	52.4	-	-	-	21.2

**Table 2:** Summary of the wear performance of the WN/MeN (Me = Cr, Zr, Mo, Nb) coatings.

Parameter	Coating System			
	WN/ZrN	WN/CrN	WN/MoN	WN/NbN
Average friction coefficient	0.76	0.52	0.47	0.55
Specific wear rate of the coating (mm^3^/Nm)	3.8 × 10^−4^	1.1 × 10^−5^	8.6 × 10^−6^	1.7 × 10^−6^
Specific wear rate of the Al_2_O_3_ ball (mm^3^/Nm)	5.9 × 10^−11^	9.6 × 10^−12^	9.9 × 10^−12^	2.0 × 10^−11^

## Data Availability

Data can be available upon request from the authors.
